# Perspectives of VA Primary Care Clinicians Toward Electronic Consultation-Related Workload Burden: A Qualitative Analysis

**DOI:** 10.1001/jamanetworkopen.2020.18104

**Published:** 2020-10-30

**Authors:** Marcie Lee, Chelsea Leonard, Preston Greene, Rachael Kenney, Melanie D. Whittington, Susan Kirsh, P. Michael Ho, George Sayre, Joseph Simonetti

**Affiliations:** 1 Denver Center of Innovation for Veteran-Centered and Value-Driven Care, VA Eastern Colorado Health Care System, Aurora; 2 Department of Health Services, University of Washington, Seattle; 3 Department of Clinical Pharmacy, University of Colorado Anschutz Medical Campus, Aurora; 4 Office of Specialty Care and Specialty Care Transformation, Washington, DC; 5 Division of Cardiology, University of Colorado School of Medicine, Aurora; 6 Seattle Center of Innovation for Veteran-Centered and Value-Driven Care, VA Puget Sound Health Care System, Seattle, Washington; 7 Division of Hospital Medicine, University of Colorado School of Medicine, Aurora

## Abstract

**Importance:**

Electronic consultation (e-consultation) is increasingly being adopted to expand access to specialty care and reduce health care costs. Little is known about clinicians’ perceptions of using e-consultations, which may be associated with program adoption.

**Objective:**

To identify perceptions of primary care clinicians in the US Veterans Health Administration (VHA) system about e-consultation and workload.

**Design, Setting, and Participants:**

A qualitative study using semistructured interviews was conducted from September 2017 through March 2018 in a national sample of VHA primary care clinics in the US. Participants were primary care clinicians who had at least 300 total patient encounters from July 2016 to June 2017, including at least 1 e-consultation request. A convenience sample of participants was recruited using email invitations. Deductive and inductive content analysis were used to identify themes. Data were analyzed from October 2017 to April 2018.

**Exposures:**

Use of e-consultation.

**Main Outcomes and Measures:**

Primary care clinician perspectives regarding e-consultation and their workload.

**Results:**

A total of 34 primary care clinicians enrolled working across 27 VHA clinical sites were included; 9 (26%) were between ages 40-49 years; 23 (68%) were female. Three themes were identified. First, the process of entering, tracking, and following up on e-consultations added a time burden to primary care clinicians. Second, e-consultation was perceived to shift diagnostic and follow-up responsibilities from specialists to primary care clinicians. Third, e-consultations were thought to improve the timeliness and quality of care provided despite a perceived increase in workload.

**Conclusions and Relevance:**

In this study, participants perceived e-consultation as valuable for patient care but also as an increase in their workload. Further work is warranted to quantify the workload increase on clinician burnout, job satisfaction, and turnover.

## Key Points

**Question:**

What are primary care clinicians’ perceptions regarding electronic consultation and their clinical workload?

**Findings:**

In this qualitative study including 34 primary care clinicians in the US Veterans Health Administration health care system, 3 themes regarding electronic consultation and workload were identified. The clinicians viewed electronic consultations as an added time burden, as shifting responsibilities from specialists to themselves, and even with the increased workload, as improving timeliness and quality of care.

**Meaning:**

The findings of this study suggest that further research is warranted to assess whether the increased workload is objective or perceived.

## Teaser

This qualitative study examines perceptions of the workload burden associated with electronic consultations among a national sample of Veterans Health Administration (VHA) primary care clinicians.

## Introduction

Health care systems are adopting electronic consultation (e-consult) programs to increase access to specialty care services.^1,2^ The Veterans Health Administration (VHA) identified access to specialty services as a leading clinical priority and adopted e-consults as one mechanism to achieve this goal.^2^ E-consultation was piloted within several VHA medical centers in 2009 and expanded nationally in 2011.^3^ However, there is substantial variation in use across VHA service regions and medical specialties.^3^ Reasons for this variation are unclear, but likely include clinician, specialty, and system-level factors.

Improving our understanding of clinician-level perceptions regarding e-consultation is important for informing program implementation and adoption, and ensuring that e-consults facilitate delivery of high-quality care. Prior evaluations demonstrated that primary care clinicians and specialists believe that e-consults may improve communication between clinicians.^4,5,6,7^ Primary care clinicians and specialists across several health care systems have reported that use of e-consultations increases the efficiency of care and reduces appointment burden for patients.^4,5,6,7^

However, little is known regarding challenges to using e-consultations, which could help explain clinician-level variation in their use. In some cases, primary care clinicians have reported that e-consultation templates are difficult to use or are inefficient.^4^ In a 2018 study, primary care clinicians working in safety-net clinics described e-consultations as “increased administrative burden, broadened clinical responsibility, and restructuring of specialty care delivery.”^8^^(pE1)^ A perception among primary care clinicians that e-consultation increases their workload may hinder adoption and lead to negative outcomes among the primary care workforce. The aim of this study was to explore perceptions regarding the association between e-consultations and workload among a national sample of VHA primary care clinicians.

## Methods

### Study Design

We conducted semistructured interviews with primary care clinicians who used e-consultations and were employed by VHA in 2017. The activities were undertaken in support of a VHA operational project and did not constitute research, in whole or in part, in compliance with VHA Handbook 1058.05, which waives the requirement for institutional review board approval.

### Participant Identification and Recruitment

We identified all VHA primary care clinicians using data from the VHA Corporate Data Warehouse. Primary care clinicians with at least 300 total patient encounters, including at least one e-consultation, between July 2016 and June 2017 were eligible for inclusion. Sites with fewer than 500 total visits, fewer than 3 specialties, or without any e-consultation visits were excluded. We enrolled a national sample of primary care clinicians because of the potential for variations in the implementation of e-consultations at the local level, and because this process was implemented across many clinical settings that likely differ with respect to their organizational climate. We sampled clinicians using a stratified purposeful strategy based on (1) clinician use of e-consultations (frequency, high or low), (2) facility use of e-consultations (frequency, high or low), (3) clinic location (Veteran Affairs Medical Center [VAMC] or community-based outpatient clinic [CBOC]), and (4) medical training (doctor vs nurse practitioner or physician assistant) (Table). We recruited and enrolled participants to balance each stratum until thematic saturation was reached within the overall sample. Primary care clinicians were recruited using email invitations followed by up to 3 reminder emails.

**Table.** Characteristics of Study Sample, by Clinician Use of e-Consultation

Characteristic	No. (%)		
	Low use (n = 18)	High use (n = 16)	Total (N = 34)
Age, y^a^			
30-39	2 (11)	1 (6)	3 (9)
40-49	3 (17)	6 (38)	9 (26)
50-59	3 (17)	4 (25)	7 (21)
>59	6 (33)	2 (13)	8 (24)
VHA career tenure, y^b^			
0-10	9 (50)	5 (31)	14 (41)
11-20	5 (28)	6 (38)	11 (32)
21-30	4 (22)	4 (25)	8 (24)
>30	0	1 (6)	1 (3)
Sex			
Male	7 (39)	4 (25)	11 (32)
Female	11 (61)	12 (75)	23 (68)
Medical training			
Advanced practice clinician (NP or PA)	6 (33)	8 (50)	14 (41)
MD or DO	12 (67)	8 (50)	20 (59)
Facility e-consultation use			
VAMC			
Low use	6 (33)	6 (38)	12 (35)
High use	6 (33)	4 (25)	10 (29)
CBOC	6 (33)	6 (38)	12 (35)
US Census region			
Northeast	2 (11)	5 (31)	7 (21)
Midwest	2 (11)	6 (38)	8 (24)
South	6 (33)	4 (25)	10 (29)
West	8 (44)	1 (6)	9 (26)

Abbreviations: CBOC, community-based outpatient clinic; DO, doctor of osteopathic medicine; MD, medical doctor; NP, nurse practitioner; PA, physician assistant; VAMC, Veteran Affairs Medical Center; VHA, Veterans Health Administration.

^a^ Age data were missing for 7 participants.

^b^ VHA career tenure is defined as the duration of years employed with VHA.

The e-consultation processes within VHA is depicted in eFigure 1 in the Supplement. An example of VHA e-consultation templates is shown in eFigure 2 in the Supplement.

### Interviews

Development of the semistructured interview guide was informed by the Practical, Robust, Implementation, and Sustainability Model (PRISM).^9,10^ Items aimed to elicit information on primary care clinicians’ experiences (including associations between e-consultations and workload), and facilitators and barriers to e-consultation use (eMethods in the Supplement).^11^ Clinicians who responded to recruitment procedures were scheduled to complete 30-minute telephone interviews with trained interviewers (C.L., M.L., P.G., and E.L.). Interviews were conducted from September 2017 through March 2018. Informed consent was obtained orally before interviews were audio recorded, deidentified, and saved on a secure server. Recordings were transcribed verbatim. Transcriptions were coded and managed in Atlas.ti version 7 (Scientific Software Development).^12^

### Qualitative Analysis

We used inductive-deductive content analysis, with predetermined code families based on barriers to using e-consultations. Inductive coding captured emergent themes. Transcripts were coded by qualitative analysts with various backgrounds, including an anthropologist (C.L.), psychologist (P.G.), social worker (R.J.), and health service researchers (M.L., E.L.). We used an iterative, team-based approach to achieve consensus. Each analyst initially independently coded the same 3 transcripts. Together, codes and emergent themes were discussed to review points of divergence and convergence and new code categories, and a refined codebook was subsequently developed. The team continued to meet and discuss new codes, update the codebook, and introduce new findings until all transcripts were coded. All quotations associated with emerging themes were reviewed to refine the theme, identify contrary cases, and assess saturation. Through further analyses and discussions, the team determined thematic saturation was reached when discussions of new notable findings waned. This study followed the Standards for Reporting Qualitative Research (SRQR) reporting guideline for qualitative studies.^10,13^

## Results

Of the 34 primary care clinicians enrolled working across 27 VHA clinical sites, 9 (26%) were between the ages of 40-49 years; 23 (68%) were female (Table). We identified 3 themes regarding the association between e-consultation and workload among primary care clinicians, including (1) the process of entering, tracking, and following up on e-consultations added time burden to primary care clinicians; (2) primary care clinicians perceived that e-consultation shifts diagnostic and follow-up responsibilities from specialists to themselves; and (3) e-consultations improve the timeliness and quality of care provided despite the perceived increase in workload.

### Time Burden

Many primary care clinicians felt that e-consultations were time consuming. Specific processes that were time consuming included using templates that were not user-friendly or that included requirements to provide patient data which they felt were not relevant for care: “To be honest, I think it’s ridiculous how many questions they ask us…I’m like, I don’t know. I have to go look that up…so why do I have to waste my time with a yes or no button…” (high user; high-use VAMC).

Others noted that templates for some specialties were “incredibly wordy,” required tangential information to complete, or required information that was not readily available in the medical record: “The [mammogram] consult…is so incredibly wordy…why am I wasting my time?” (high user; high-use VAMC).

When medical information was not readily accessible, primary care clinicians reported using appointment time to navigate patients’ medical records: “They expect you to put in all of the physical findings and exams. I’m like, give me a break, in 30 minutes, if I were to do that… I can’t be doing their diabetes, their hypertension, their cholesterol and everything, so that’s where, you know, sometimes I get the burden of being a primary care physician, where I’m expected to do all the initial work for the specialist” *(*high user; high-use VAMC).

In some cases, the time burden required to place e-consultations forced primary care clinicians to complete them outside of allotted appointment times and experienced challenges in transferring information from the patient medical record to the e-consultation template: “Well, the ones I don’t like is when I’m in the middle of trying to do it and they’re asking all these questions that I don’t have the answers to, and I can’t get back to the chart” (low user; low-use VAMC).

Tracking e-consultations added to primary care clinicians’ workload, and sometimes made it difficult to close the communication loop with the specialist and patient: “I think the biggest downside for e-consults…is that…instead of having them be seen by…a specialist, you get sort of recommendations back that then require a lot more work on your part in terms of ordering tests, following up with the patient, communicating things, and I think that workload shift from specialty to primary care is my biggest barrier” (low user; high-use VAMC).

In some cases, primary care clinicians felt that specialists did not respond to e-consultations in a timely manner, or e-consultations were discontinued requiring the primary care clinician to re-enter the consultation. One primary care clinician described the burden of tracking e-consultations: “I think (e-consults) being discontinued and then re-entering it is just needless work…it falls on me to go and enter, click all the buttons… and then I have to make sure that I see all the consults and have to be alerted because if doctor makes a comment that tells me to do something, [and] if I'm not getting all the alerts, I'll miss stuff... you have to see every little note, patient scheduled, talked to patient, patient no-showed, and you’re getting every single alert because you never know which ones are pertinent…so it increases the workload of alerts that you have to review” (high user; high-use VAMC).

Similarly, another clinician said: “(My e-consultations) could go out to potentially up to 3 months of chart review which is beyond acceptable… so that has generated a mechanism whereby I must find some sort of tracking system so those people are not forgotten” (high user; low-use VAMC).

Many primary care clinicians described a risk of losing track of the e-consultation if specialists did not direct their response specifically to them: “…the return response really should come by way of an additional view alert… they’ll respond back and forget to tag us on, and then it would be so easy to forget that you’ve either put it in or further not know that it had been responded to…and only serendipitously would you find the response” (high user; low-use VAMC).

### Increased Responsibility

Participants reported that using e-consultations resulted in a shift of work from specialists to themselves. Specialists often recommended that primary care clinicians obtain diagnostic testing that they would typically not be responsible for ordering or interpreting. In some cases, primary care clinicians did not have the approval to order specialized tests: “…they want us to order testing that is not appropriate for primary care… I’m not the one to order a CT myelogram…Yes, you want an MRI, it’s a different story, but a CT myelogram, what am I supposed to do with it, to put another consult to them?” (high user; high-use VAMC).

Some primary care clinicians were uncomfortable with the responsibility of ordering and being responsible for specialized tests: “… I’m working up someone with potential leukemia, (and) ordering all these diagnostic tests, some of which, it’s not within my scope of practice to interpret…So, that’s a little bit stressful, the whole idea of doing some of this workup that’s recommended through e-consults and not being educated exactly on what it means or what I’m doing, that kind of increases the workload” (low user; CBOC).

One clinician seemed to feel that this shift of responsibility has become normalized: “I have a patient… [with] chronic anemia… I pretty much do some of the basic workup...and I ask them, what more do I still need to do? That’s when I get the input from them saying, OK, do this mutation or send a special, peripheral blood smear, and they would tell me who to send it to. That’s a little extra work for us, but I guess that’s how it is” *(*high user; high-use VAMC).

In some instances, there was uncertainty in who was responsible for ordering tests: “…they’ll say they want labs… I don’t know if they sent them to the lab there, or if they’re gonna come back to [me] and I’m supposed to do it” (high user; CBOC). One primary care clinician said, “they would just sort of wash their hands of the problem and make it somebody’s else’s problem” (high user; CBOC).

### Electronic Consultation Value

Despite the increased workload, primary care clinicians expressed that e-consultations were generally effective at coordinating timely care without trade-offs to quality or continuity: “I do get a more timely response…rather than having to have the veteran wait for a week or 2 weeks or sometimes 4 weeks to get in with the specialist” (low user; CBOC).

E-consultation has alleviated some primary care clinician concerns about the uncertainty of patients following through with in-person appointments because the “person doesn’t have to come back and forth to multiple appointments” (high user; low-use VAMC). Several clinicians reported that e-consultations have reduced unnecessary specialty appointments and patient travel. In addition, 1 primary care clinician felt that the additional workload carried by primary care clinicians could cut down the work for specialists, who he perceived as overworked: “It’s added to the primary provider…but it’s cutting down on our… overworked specialists… when we don’t have the money or we can’t pay them enough to come, and they’ve got a huge amount of work on their plate…” (low user, low-use VAMC).

Patient satisfaction, quality of care, and increasing access to specialty care that would otherwise be unavailable were reasons primary care clinicians continued using e-consultations despite their increased workload: “E-consults have been very good for the patient. Patients are happier and they improve patient care and patient safety. The e-consult process has made more work for us…but on the other hand, before we had them… you just had no one else to help you with it, so it’s taken some pressure off me and I think the patients are getting better care, but it’s more work” (high user; CBOC).

## Discussion

In this study of a national sample of VHA primary care clinicians who use e-consultation, most perceived e-consultations as increasing their clinical workload. We identified specific mechanisms by which e-consultations added to their workload, including time needed to enter and track consultations, and shifting responsibility for the diagnosis and management of conditions that would typically fall outside their scope of practice. That e-consultation may increase clinical workload among primary care clinicians has several important implications for VHA and other health care systems which have prioritized this mechanism of care delivery.

A perception that e-consultations create additional work may hinder their adoption and use.^11,14^ Prior studies have already shown that primary care clinicians have limited time to complete their clinical tasks, which has been exacerbated by increases in patient complexity, new responsibilities related to team-based care, and technologic advances (eg, e-consultations).^15,16^ Additional studies among clinicians who have abandoned this technology or have yet to adopt it may be helpful in improving our understanding of these challenges.

An important question is what trade-offs are occurring to allow for this increase in workload. For example, it is unknown whether clinicians are avoiding other recommended clinical tasks (eg, dietary counseling) to allow for the completion and tracking of e-consultations. If clinicians are completing e-consultation requests during clinical encounters, this process may reduce the amount of time they are able to spend in face-to-face communication with their patients, which may have an adverse effect on patient satisfaction.^17^ Alternatively, primary care clinicians may adapt to this workload change by increasing work hours or completing work during nonclinical time. This trade-off could have important implications for the primary care workforce given the high prevalence of burnout among VHA primary care staff,^18^ and that burnout is associated with staff absenteeism^19^ and turnover.^20^

An important question is whether the increased workload reported by primary care clinicians in this sample is objective or perceived, and how specialists consider e-consultations to affect their workload. Prior studies revealed that specialists report time constraints in completing consultations, in addition to increased e-consultation volumes over time.^1,21^ One study found that specialists perceived that 27% of their e-consultations were new work.^2^ That is, those consultations would not have occurred in the absence of an e-consultation mechanism.^22^ Additional studies using survey or time-in-motion methodology could be helpful in validating clinicians’ perceptions about workload. If e-consultation is adding to workload among primary care clinicians or specialists, the VHA and other health care systems may need to consider how to account for that work within productivity requirements and reimbursement plans.

We identified several practical ways in which primary care clinicians’ concerns could potentially be addressed. First, most clinicians described ways in which templates could facilitate or hinder e-consultation. Future work could aim to develop specialty-specific high-quality templates that facilitate efficient and effective communication between clinicians. Clinicans in this sample also reported several communication challenges, including confusion regarding test ordering and interpretation responsibilities, and communication with patients. Facility-specific and specialty-specific service agreements developed in a collaborative manner may be useful in addressing these issues. Consistent with this approach, the VHA has developed a clinician toolkit which outlines processes by which services and clinical sites might coordinate e-consultation procedures.^22^

Several participants reported concerns about tracking e-consultations and ways in which responses could be lost. Primary care clinicians described developing workarounds to manage this, such as creating their own tracking system to ensure completion of e-consultations. These workarounds were also described as contributing to their workload, and it is unknown how effective they may be in preventing adverse events (eg, test results lost to follow-up). Prior studies illustrated that clinical workarounds, although potentially necessary when clinicians are faced with unintended challenges, are generally suboptimal and may jeopardize patient safety, efficiency, and effectiveness of care.^23^

Despite these challenges, primary care clinicians generally agreed that e-consultations improved quality and access to care. This agreement is consistent with other studies among VHA and non-VHA clinicians, which have demonstrated improved timeliness for specialty care input, reduced unnecessary face-to-face visits, improved clinician communications and patient care, and that e-consultations have an educational benefit for clinicians.^2,8,21,24,25,26^

This study was conducted within the VHA. Thus, some findings (eg, frustration with e-consultation templates) may be specific to the VHA. However, the main finding of this study is consistent with an abundance of organizational function literature demonstrating that new processes and technology often have adverse and unexpected effects on clinicians.^2,8,21,24,25,26^ Other health care systems may need to consider assessing the extent to which implementing e-consultation may shift clinical responsibilities among clinicians, and develop strategies to mitigate or account for that shift.

### Limitations

This study has limitations. The generalizability of findings is limited given the qualitative study design, and we enrolled a small sample of clinicians working in VHA clinics. Although we used several methods to limit interpreter bias, the identified themes could have been affected by interpreter bias. Similarly, participants’ reports of experiences using e-consultations are subject to recall and social desirability biases.

## Conclusions

To our knowledge, this study provides the first insights into perceptions of workload associated with e-consultation among a national sample of clinicians. That many primary care clinicians feel that e-consultations increase their workload has implications for the future use of this technology and the well-being of patients and clinicians. Additional work is warranted to quantify the potential time burden reported by primary care clinicians, capture specialists’ perspectives, and assess workload increases with regard to clinician outcomes such as burnout and turnover.

## Notes

Supplement. **eFigure 1.** Overview of VHA e-Consultation Process **eFigure 2.** Overview of VHA e-Consultation Template **eMethods.** Interview Guide
