# Supplementary material for: Perspectives of VA Primary Care Clinicians Toward Electronic Consultation-Related Workload Burden: A Qualitative Analysis
Source: JAMA Netw Open. 2020 Oct 30;3(10):e2018104. doi: 10.1001/jamanetworkopen.2020.18104 (PMC7599439; doi:10.1001/jamanetworkopen.2020.18104)
Supplement: Supplement. — eFigure 1. Overview of VHA e-Consultation Process eFigure 2. Overview of VHA e-Consultation Template eMethods. Interview Guide [file jamanetwopen-e2018104-s001.pdf]

# Supplemental Online Content

Lee M, Leonard C, Greene P, et al. Perspectives of VA primary care clinicians toward electronic consultation-related workload burden: a qualitative analysis. *JAMA Netw Open*. 2020;3(10):e2018104. doi:10.1001/jamanetworkopen.2020.18104

**eFigure 1.** Overview of VHA e-Consultation Process

**eFigure 2.** Overview of VHA e-Consultation Template

**eMethods.** Interview Guide

This supplemental material has been provided by the authors to give readers additional information about their work.

© 2020 Lee M et al. *JAMA Network Open*.

eFigure 1. **Overview of VHA e-consultation process**

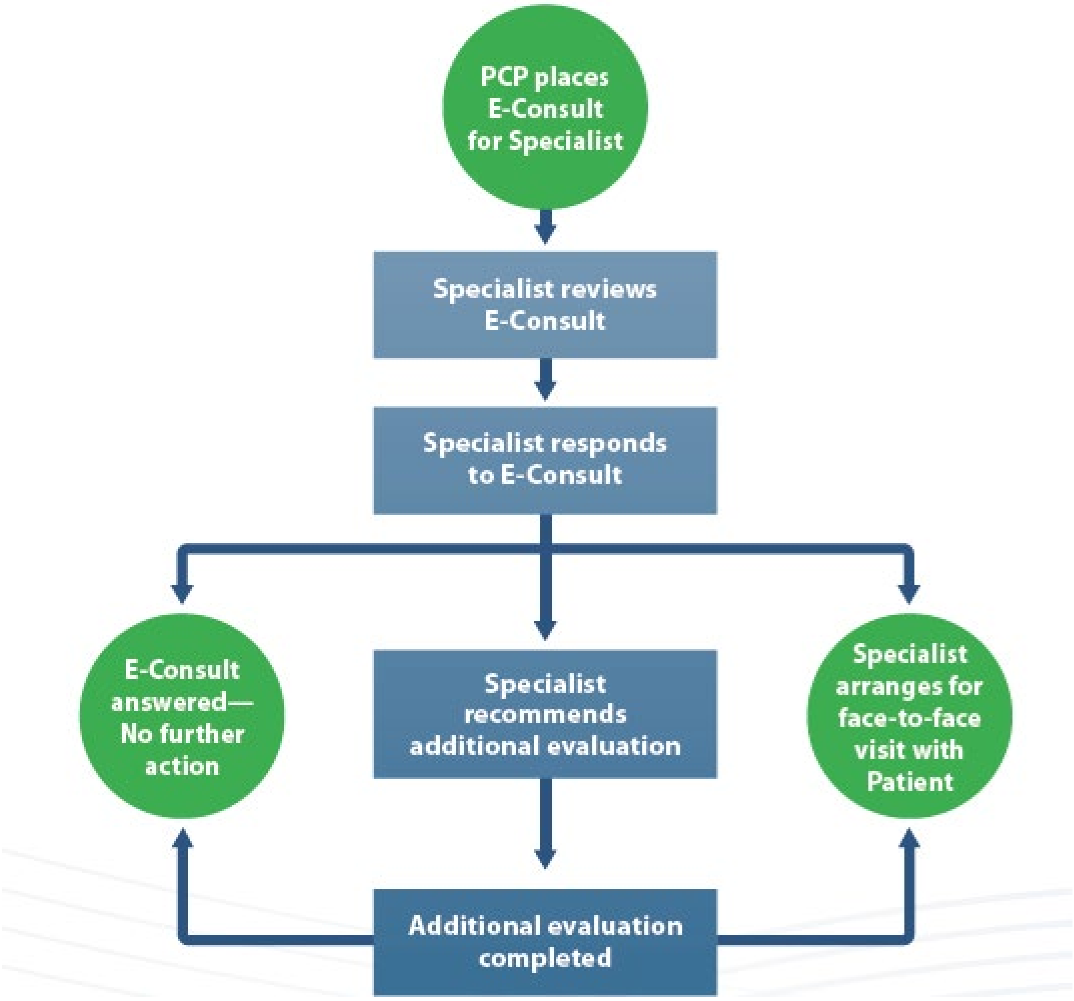


© 2020 Lee M et al. *JAMA Network Open*.

eFigure 2. **Overview of VHA e-consultation template**

The template automatically pulls in relevant lab and imaging data for the subcategory of consult being placed. For example, an endocrinology e-consult for evaluation of suspected pituitary tumor includes brain MRI and pituitary-related labwork.

```
E-consult: Provider-to-provider endocrine consultation with response
           within 48 hours.

  <-- Click here to begin!
      Select the reason for your consult:

    Thyroid
    Pituitary
        <--- Click here to continue!
        Diagnosis:*
        tumor
        insufficiency
        other

        * New diagnosis  Prior diagnosis

        Records including imaging available: * CPRS  VistA  Outside Facility  Unknown

        Pertinent Labs:
          No INSULIN-LIKE GROWTH FACT.I (LC010363) in the last 6M
          No GROWTH HORMONE (LC004275) in the last 6M
          TSH - NONE FOUND
          No T-4  in the last 6M
          No TRIIODOTHYRONINE, TOTAL (LC002188) QQQ in the last 6
          No LUTEINIZING HORMONE in the last 6M
          No FSH in the last 6M
          No TESTOSTERONE (DVAMC) in the last 6M
          No ESTRADIOL (LC004515) QQQ in the last 6M
          No ACTH (ON-ICE) (LC004440) QQQ in the last 6M
          No CORTISOL in the last 6M
          No PROLACTIN in the last 6M
          SODIUM - NONE FOUND
          POTASSIUM - NONE FOUND
          CHLORIDE - NONE FOUND
          CO2 - NONE FOUND
          CREATININE SERUM - NONE FOUND
          No GLUCOSE in the last 6M

        Pertinent Imaging:
        No data available for ZZMRI BRAIN; ZZMRI BRAIN W/CONTRAST;
      MRI BRAIN W/WO GADOLINIUM; ZZMRI BRAIN WO THEN W/CONTRAST
```

© 2020 Lee M et al. *JAMA Network Open*.

eMethods. **E-Consults Interview Guide**

*Interviewer Name:*

*Interview date:* *Initial interview date:*

*Start time:* *End time:*

Hello [Dr./Mr./Ms. interview participant name],

My name is [interviewer name]. I am with an evaluation team tasked with understanding how E-Consults are used in [site].

These findings will be used to understand barriers and facilitators to E-consult utilization and expansion, and to develop 'best practices' recommendations and support tools to facilitate E-consult expansion. Your responses will be kept confidential and you and your facility will not be identified in any reports or publications. Nothing that you say will be reported back to your facility.

The call will take approximately 20 to 30 minutes.

Your participation in this interview is voluntary. You can stop the interview at any time and let us know if you'd rather not answer a particular question.

Do you have any questions?

In order to make sure we capture all of the information you give us, we would like to record this call. The audio-file for the recording will be uploaded to a restricted access file on the VA intranet immediately after we complete this interview. The audio file will be saved anonymously. We may transcribe the recording, and your name will be removed from any transcripts. Is this okay with you?

*Grounded prompts: If responses are limited or require clarification, probes may be used to illicit more detailed responses. Probes should use words or phrases presented by the participant using one of the following formats:*
*1. What do you mean by ____________?*
*2. Tell me more about ____________.*
*3. Give me an example of ____________.*
*4. Tell me about a time when ____________.*
*5. Who __________?*
*6. When __________?*

1. Please tell me about your role with e-consults.

2. Please tell me about E-consults.
   *(PRISM: Organizational Perspective)*

3. What, if any, are challenges to using E-consults at your site?
   *(PRISM: Organizational Perspective)*
   a. Do you have any suggestions for overcoming these barriers?

4. (If needed) What is a good E-Consult?
   a. Grounded probes- probe for specific examples

5. What, if anything, has made it easier to use E-consults at your site?
   *(PRISM: Organizational Perspective)*

6. How have E-Consults affected your workload?
   *(PRISM: Organizational Perspective)*
   a. How do E-Consults fit into your practice?

7. How have E-Consults affected communication between PCPs and specialists?

© 2020 Lee M et al. *JAMA Network Open*.

*(PRISM: Organizational Perspective)*

8. How do you think E-Consults have affected the quality of care provided to patients?
   *(PRISM: Patient Perspective)*
   a. Can you give me an example?

9. What types of patients do you use E-Consults for?
   *(PRISM: Characteristics of Organizational Recipients)*
   a. Who were these people?
   b. Can you give me an example?

10. What kind of communication or feedback do you receive from your division or facility leadership about your use of E-Consults?
    a. Can you tell me about how it was rolled out?
    b. Are there expectations for e-consult use?
11. How have e-consults been used to coordinate care or procedures for your patients? – probe for specific examples

12. Is there anything else you would like us to know about the use of E-Consults at your site?

13. Do you have any advice on E-Consults for other sites and/or specialties?

    a. Probe about templates

*Thank you for that information about the E-Consult program.*

14. Do you have any questions for us?

Thank you for participating in this interview.

© 2020 Lee M et al. *JAMA Network Open*.
